# Supplementary material for: Seminoma and Embryonal Carcinoma Footprints Identified by Analysis of Integrated Genome-Wide Epigenetic and Expression Profiles of Germ Cell Cancer Cell Lines
Source: PLoS One. 2014 Jun 2;9(6):e98330. doi: 10.1371/journal.pone.0098330 (PMC4041891; doi:10.1371/journal.pone.0098330)
Supplement: File S1 — ZIP file containing DMRforPairs output for significant regions. Please start from the html files. (ZIP) [file pone.0098330.s008.zip › figures/7242.pdf]

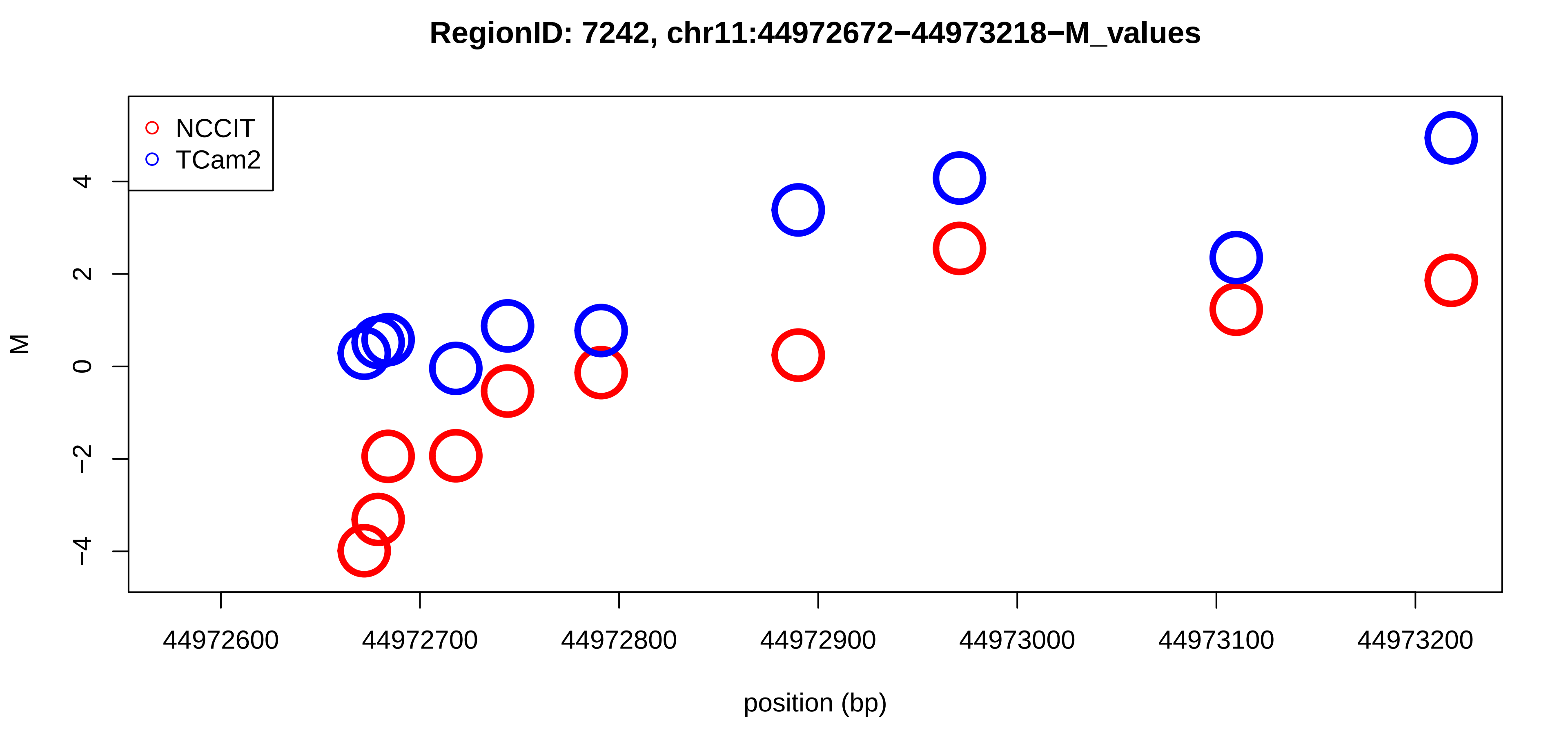
RegionID: 7242, chr11:44972672−44973218−M_values
NCCIT
TCam2
M
position (bp)
44972600
44972700
44972800
44972900
44973000
44973100
44973200
−4
−2
0
2
4

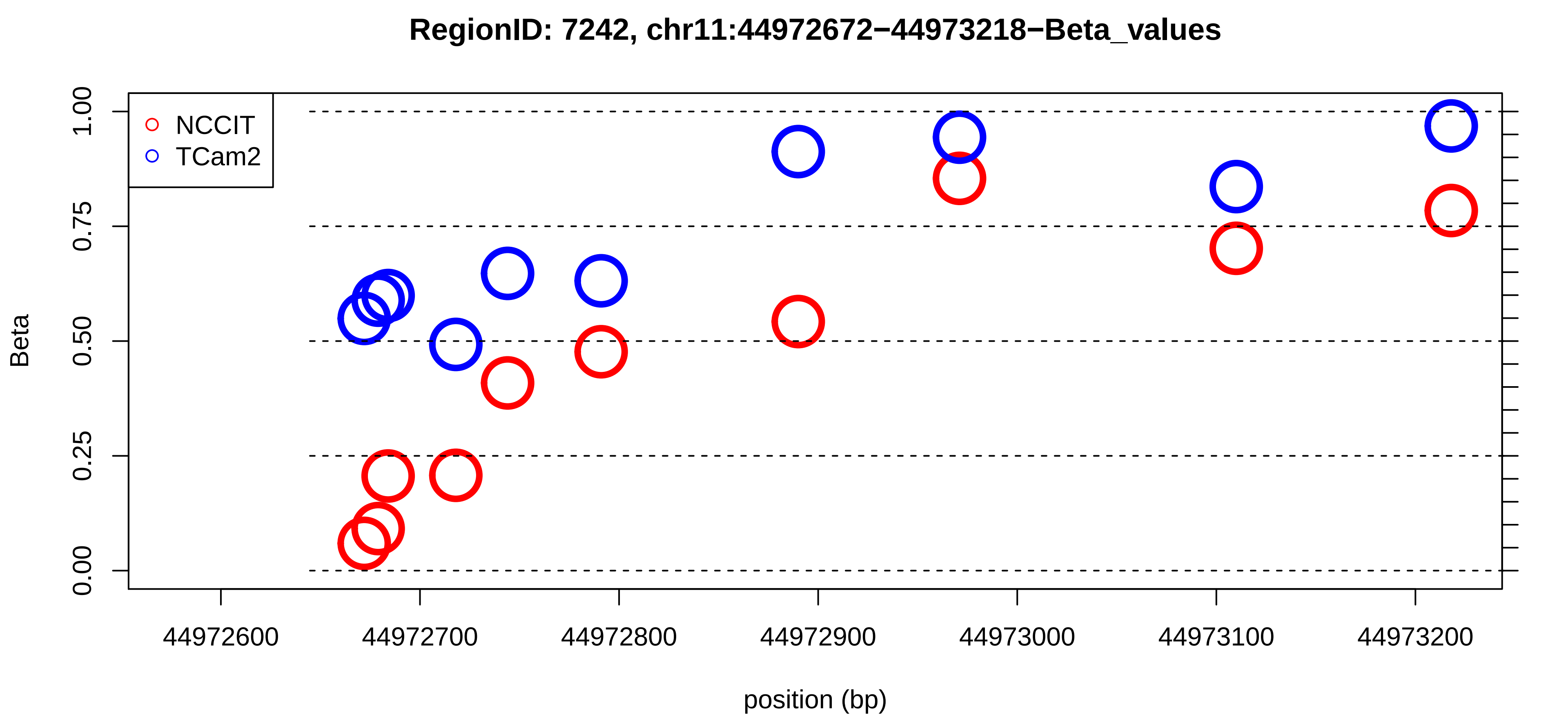
RegionID: 7242, chr11:44972672−44973218−Beta_values
NCCIT
TCam2
Beta
position (bp)
44972600
44972700
44972800
44972900
44973000
44973100
44973200
0.00
0.25
0.50
0.75
1.00
